# Supplementary material for: Diagnosis and prognosis prediction of gastric cancer by high-performance serum lipidome fingerprints
Source: EMBO Mol Med. 2024 Nov 14;16(12):3089–112. doi: 10.1038/s44321-024-00169-0 (PMC11628598; doi:10.1038/s44321-024-00169-0)
Supplement: Supplementary file 1 — Table EV1 [file 44321_2024_169_MOESM1_ESM.docx]

**Table EV1. The attributes of the 19 metabolites in the SLMS.**

| metabolites | ESI pattern | coefficient | Formula | Oncology | Average m/z |
| --- | --- | --- | --- | --- | --- |
| LPC 17:0 | positive | -1.165 | C_25_H_52_NO_7_P | LPC | 510.36 |
| PE O-44:6  \|PE O-24:2_20:4 | positive | 0.151 | C_49_H_88_NO_7_P | EtherPE | 834.63 |
| CAR 14:0 | positive | -0.446 | C_21_H_41_NO_4_ | CAR | 372.31 |
| HexCer 42:2;2O  \|HexCer 18:1;2O/24:1 | positive | 0.085 | C_48_H_91_NO_8_ | HexCer | 792.67 |
| FA 28:3;O | negative | -0.078 | C_28_H_50_O_3_ | OxFA | 433.36 |
| HexCer 42:2;3O | negative | -0.223 | C_48_H_91_NO_9_ | HexCer | 884.68 |
| LPE O-14:1 | negative | 0.676 | C_19_H_40_NO_6_P | EtherLPE | 408.25 |
| PE 36:4\|PE 18:2_18:2 | negative | -0.218 | C_41_H_74_NO_8_P | PE | 738.51 |
| CAR 18:1 | negative | 0.329 | C_25_H_47_NO_4_ | CAR | 426.36 |
| Cer 38:1;2O  \|Cer 18:1;2O/20:0 | negative | 0.262 | C_38_H_75_NO_3_ | Cer | 652.58 |
| TG(P) 50:2 | positive | 0.130 | C_53_H_100_O_5_ | TG | 834.79 |
| FA 18:0;O | negative | -0.469 | C_18_H_36_O_3_ | OxFA | 299.26 |
| PC O-38:3 | positive | -0.019 | C_46_H_88_NO_7_P | EtherPC | 798.64 |
| FA 16:2 | negative | 0.144 | C_16_H_28_O_2_ | FA | 251.20 |
| PC O-42:6  \|PC O-22:2_20:4 | positive | -0.019 | C_50_H_90_NO_7_P | EtherPC | 848.64 |
| Hex2Cer 42:2;2O  \|Hex2Cer 18:1;2O/24:1 | positive | 0.150 | C_54_H_101_NO_13_ | HexCer | 972.73 |
| PE 40:7\|PE 18:1_22:6 | negative  positive | 0.129 | C_45_H_76_NO_8_P | PE | 788.51 |
| LPE 20:4 | positive | 0.449 | C_25_H_44_NO_7_P | LPE | 502.29 |
| PE 40:3 | negative | 0.068 | C_45_H_84_NO_8_P | PE | 796.58 |

**Legend**: CAR, acyl carnitine; Cer, ceramide; ESI, electron spray ionization; EtherLPE, ether-linked lyso-phosphatidylethanolamine; EtherPC, ether-linked phosphatidylcholine; EtherPE, ether-linked phosphatidylethanolamine; FA, fatty acid; HexCer, hexosylceramide; LPC, lyso-phosphatidylcholine; LPE, lyso-phosphatidylethanolamine; OxFA, oxidized fatty acid; PE, phosphatidylethanolamine; SLMS, serum lipid metabolic signature; TG, triglyceraide.
