# Supplementary material for: Diagnosis and prognosis prediction of gastric cancer by high-performance serum lipidome fingerprints
Source: EMBO Mol Med. 2024 Nov 14;16(12):3089–112. doi: 10.1038/s44321-024-00169-0 (PMC11628598; doi:10.1038/s44321-024-00169-0)
Supplement: Supplementary file 2 — Table EV2 [file 44321_2024_169_MOESM2_ESM.docx]

**Table EV2. Analyses of the impact of clinical characteristics on the lipidomics data of the LDA-aid SLMS in the training cohort.**

| Types | Factors | Univariate analysis | | | | | | Multivariate analysis | | | | | |
| --- | --- | --- | --- | --- | --- | --- | --- | --- | --- | --- | --- | --- | --- |
|  |  | Standardized  Coefficient | | | 95%CI | *P* value^a^ | | Standardized  Coefficient | | | 95%CI | *P* value^a^ | |
| Gastric cancer | Sex | | -0.46 | -0.68~1.69 | | | 0.43 | | 1.02 | -0.59~3.06 | | | 0.28 |
|  | Age | | 0.03 | -0.01~0.08 | | | 0.17 | | 0.04 | -0.02~0.09 | | | 0.18 |
|  | CEA | | 0.12 | -0.01~0.59 | | | 0.47 | | 0.08 | -0.05~0.58 | | | 0.67 |
|  | CA19-9 | | 0.00 | -0.00~NA | | | 0.77 | | 0.00 | -0.00~NA | | | 0.88 |
|  | CA72-4 | | 0.02 | -0.00~0.19 | | | 0.66 | | 0.01 | -0.00~0.17 | | | 0.84 |
|  | Maximum diameter | | 0.02 | -0.01~0.06 | | | 0.25 | | 0.00 | -0.03~0.04 | | | 0.78 |
|  | pTNM | | 0.38 | -0.19~1.00 | | | 0.20 | | -0.10 | -1.06~0.94 | | | 0.85 |
|  | Differentiation | | 0.45 | -1.11~1.66 | | | 0.51 | | 0.73 | -0.96~2.25 | | | 0.36 |
|  | Vascular invasion | | 0.74 | -0.43~2.15 | | | 0.25 | | 0.29 | -1.16~1.86 | | | 0.69 |
|  | Nerve infiltration | | 0.75 | -0.39~1.94 | | | 0.20 | | 0.69 | -1.31~2.70 | | | 0.50 |
|  | Smoking history | | -0.16 | -1.37~1.29 | | | 0.81 | | -1.65 | -4.01~0.48 | | | 0.14 |
|  | Drinking history | | 0.55 | -0.93~2.79 | | | 0.54 | | 1.28 | -0.81~3.89 | | | 0.27 |
|  | HER2 | | -0.12 | -1.60~1.42 | | | 0.88 | | 0.10 | -1.53~1.80 | | | 0.91 |
|  | Family tumor history | | -0.52 | -1.81~1.08 | | | 0.46 | | -0.34 | -1.72~1.33 | | | 0.65 |
|  | BMI | | -0.03 | -0.22~0.16 | | | 0.72 | | -0.05 | -0.26~0.15 | | | 0.61 |
| Healthy  Donors | Sex | | -0.50 | -1.86~0.75 | | | 0.44 | | -0.40 | -1,81~0.92 | | | 0.56 |
|  | Age | | -0.01 | -0.07~0.03 | | | 0.58 | | -0.01 | -0.06~0.04 | | | 0.73 |
|  | CEA | | -0.19 | -0.85~0.25 | | | 0.52 | | -0.13 | -0.85~0.30 | | | 0.67 |
|  | CA19-9 | | 0.00 | -0.09~0.06 | | | 0.98 | | 0.00 | -0.09~0.06 | | | 0.96 |
|  | CA72-4 | | 0.00 | -0.16~0.07 | | | 0.99 | | 0.00 | -0.16~0.07 | | | 0.99 |

**Legend**: BMI, body mass index; CA19-9, carbohydrate antigen 199; CA72-4, carbohydrate antigen 724; CEA, carcinoembryonic antigen; CI, confidence interval; HER2, human epidermal growth factor receptor 2; pTNM, pathological, tumor, node, metastasis; SLMS, serum lipid metabolic signature.

Dependent variable: scores of SLMS.

^a^Wald Test was used to calculate *P* value.
