# Supplementary material for: Diagnosis and prognosis prediction of gastric cancer by high-performance serum lipidome fingerprints
Source: EMBO Mol Med. 2024 Nov 14;16(12):3089–112. doi: 10.1038/s44321-024-00169-0 (PMC11628598; doi:10.1038/s44321-024-00169-0)
Supplement: Supplementary file 3 — Table EV3 [file 44321_2024_169_MOESM3_ESM.docx]

**Table EV3. Analyses of the impact of clinical characteristics and SLMS on the distribution of lipidomics data in the training set.**

| Factors | Univariate analysis | | | | | | Multivariate analysis | | | | |
| --- | --- | --- | --- | --- | --- | --- | --- | --- | --- | --- | --- |
|  | Standardized  Coefficient | | | 95%CI | *P* value^a^ | | Standardized  Coefficient | | | 95%CI | *P*  value^a^ |
| Sex | | -0.04 | -0.40~0.33 | | | 0.85 | | 0.12 | -1.16~1.42 | | 0.85 |
| Age | | -0.002 | -0.02~0.01 | | | 0.79 | | -0.01 | -0.06~0.04 | | 0.66 |
| CEA | | 0.06 | -0.00~0.15 | | | 0.17 | | 0.02 | -0.03~0.40 | | 0.90 |
| CA19-9 | | 0.01 | 0.00~0.02 | | | 0.06 | | 0.00 | -0.00~0.05 | | 0.81 |
| CA72-4 | | 0.01 | -0.00~0.03 | | | 0.21 | | 0.00 | -0.01~NA | | 0.98 |
| SLMS | | 8.56 | 7.18~10.31 | | | <0.001 | | 8.52 | 7.13~10.30 | | <0.001 |

**Legend**: CA19-9, carbohydrate antigen 199; CA72-4, carbohydrate antigen 724; CEA, carcinoembryonic antigen; CI, confidence interval; SLMS, serum lipid metabolic signature.

Dependent variable: sample status.

^a^Wald Test was used to calculate *P* value.
