# Supplementary material for: Diagnosis and prognosis prediction of gastric cancer by high-performance serum lipidome fingerprints
Source: EMBO Mol Med. 2024 Nov 14;16(12):3089–112. doi: 10.1038/s44321-024-00169-0 (PMC11628598; doi:10.1038/s44321-024-00169-0)
Supplement: Supplementary file 4 — Table EV4 [file 44321_2024_169_MOESM4_ESM.docx]

**Table EV4. The performance of SLMS when using in comparing any two groups in the predictive cohort.**

| Predictive cohort | Accuracy(95%CI) | Sensitivity(95%CI) | Specificity(95%CI) |
| --- | --- | --- | --- |
| GC vs HD | 0.938  (0.885~0.971) | 0.934  (0.853~0.978) | 0.942  (0.858~0.984) |
| PL vs HD | 0.771  (0.693~0.838) | 0.606  (0.483~0.720) | 0.942  (0.858~0.984) |
| GC vs PL | 0.673  (0.591~0.749) | 0.934  (0.853~0.978) | 0.394  (0.280~0.518) |

**Legend**: CI, confidence interval; GC, gastric cancer; HD, healthy donor; PL, precancerous lesion.
