# Supplementary material for: Diagnosis and prognosis prediction of gastric cancer by high-performance serum lipidome fingerprints
Source: EMBO Mol Med. 2024 Nov 14;16(12):3089–112. doi: 10.1038/s44321-024-00169-0 (PMC11628598; doi:10.1038/s44321-024-00169-0)
Supplement: Supplementary file 5 — Table EV5 [file 44321_2024_169_MOESM5_ESM.docx]

**Table EV5. The performance of SLMS in distinguishing GC patients with different pTNM stages from healthy donors.**

| Cohort | Stage  (N) | AUC | Accuracy  (95%CI) | Sensitivity  (95%CI) | Specificity  (95%CI) |
| --- | --- | --- | --- | --- | --- |
| Training cohort | I (73) | 0.990 | 0.963  (0.935~0.982) | 0.932  (0.847~0.977) | 0.974  (0.943~0.990) |
|  | II (36) | 0.998 | 0.973  (0.946~0.989) | 0.972  (0.855~0.999) | 0.974  (0.943~0.990) |
|  | III (100) | 0.994 | 0.973  (0.948~0.987) | 0.970  (0.915~0.994) | 0.974  (0.943~0.990) |
|  | IV (18) | 0.999 | 0.975  (0.948~0.991) | 1.000  (0.815~1.000) | 0.974  (0.943~0.990) |
| Testing cohort | I (13) | 1.000 | 0.980  (0.897~1.000) | 0.923  (0.640~0.998) | 1.000  (0.910~1.000) |
|  | II (11) | 1.000 | 1.000  (0.929~1.000) | 1.000  (0.715~1.000) | 1.000  (0.910~1.000) |
|  | III (13) | 1.000 | 1.000  (0.932~1.000) | 1.000  (0.753~1.000) | 1.000  (0.910~1.000) |
|  | IV (2) | 0.795 | 0.976  (0.871~0.999) | 0.500  (0.013~0.987) | 1.000  (0.910~1.000) |
| External validation cohort | I (23) | 0.962 | 0.909  (0.843~0.954) | 0.957  (0.781~0.999) | 0.898  (0.820~0.950) |
|  | II (20) | 0.989 | 0.915  (0.850~0.959) | 1.000  (0.832~1.000) | 0.898  (0.820~0.950) |
|  | III (47) | 0.969 | 0.903  (0.843~0.946) | 0.915  (0.796~0.976) | 0.898  (0.820~0.950) |
|  | IV (8) | 0.892 | 0.887  (0.811~0.940) | 0.750  (0.349~0.968) | 0.898  (0.820~0.950) |
| Predictive cohort | I (20) | 0.944 | 0.921  (0.845~0.968) | 0.850  (0.621~0.968) | 0.942  (0.858~0.984) |
|  | II (15) | 0.987 | 0.941  (0.867~0.980) | 0.933  (0.681~0.998) | 0.942  (0.858~0.984) |
|  | III (33) | 0.988 | 0.951  (0.889~0.989) | 0.970  (0.842~0.999) | 0.942  (0.858~0.984) |
|  | IV (8) | 1.000 | 0.948  (0.872~0.986) | 1.000  (0.631~1.000) | 0.942  (0.858~0.984) |

**Legend**: SLMS, serum lipid metabolic signature; GC, gastric cancer; AUC, area under curve; CI, confidence interval.
