# Supplementary material for: Diagnosis and prognosis prediction of gastric cancer by high-performance serum lipidome fingerprints
Source: EMBO Mol Med. 2024 Nov 14;16(12):3089–112. doi: 10.1038/s44321-024-00169-0 (PMC11628598; doi:10.1038/s44321-024-00169-0)
Supplement: Supplementary file 6 — Table EV6 [file 44321_2024_169_MOESM6_ESM.docx]

**Table EV6. The two-year overall survival of patients with different prognostic subtypes in each cohort.**

|  | SI  (two-year overall survival*) | SII  (two-year overall survival*) |
| --- | --- | --- |
| Exploration cohort | 75.7 (67.6~84.7) | 88.8(83.8~94.0) |
| External validation cohort | 59.1 (45.5~76.7) | 89.2(81.4~97.8) |
| Predictive cohort | 58.6 (40.1~84.1) | 91.4(83.7~99.9) |

**Legend**: *Data are presented as % (95% confidence interval).
