# Supplementary material for: Diagnosis and prognosis prediction of gastric cancer by high-performance serum lipidome fingerprints
Source: EMBO Mol Med. 2024 Nov 14;16(12):3089–112. doi: 10.1038/s44321-024-00169-0 (PMC11628598; doi:10.1038/s44321-024-00169-0)
Supplement: Supplementary file 7 — Table EV7 [file 44321_2024_169_MOESM7_ESM.docx]

**Table EV7. Overlap between the prognostic subtypes and clinicopathologic characteristics.**

| Characteristics | Exploration  cohort | | | External validation  cohort | | | | Predictive  cohort | | |
| --- | --- | --- | --- | --- | --- | --- | --- | --- | --- | --- |
|  | SI | SII | *P* value^a^ | SI | SII | | *P* value^a^ | SI | SII | *P* value^a^ |
| Age(year) |  |  | 1.000 |  |  | | 0.776 |  |  | 1.000 |
| <=40 | 19 | 29 |  | 5 | 5 | |  | 0 | 0 |  |
| >40 | 84 | 134 |  | 35 | 53 | |  | 23 | 53 |  |
| Sex |  |  | 0.955 |  |  | | 0.040 |  |  | 0.604 |
| female | 50 | 81 |  | 20 | 16 | |  | 9 | 13 |  |
| male | 53 | 82 |  | 20 | 42 | |  | 17 | 37 |  |
| Maximum diameter(mm) |  |  | 0.001 |  |  | | 0.012 |  |  | <0.001 |
| <=50 | 73 | 143 |  | 23 | 48 | |  | 12 | 45 |  |
| >50 | 30 | 19 |  | 17 | 10 | |  | 13 | 45 |  |
| Vascular invasion |  |  | 0.014 |  |  | | 0.176 |  |  | 0.285 |
| no | 48 | 101 |  | 22 | 41 | |  | 10 | 29 |  |
| yes | 55 | 60 |  | 17 | 16 | |  | 14 | 21 |  |
| Nerve infiltration |  |  | 0.010 |  |  | | 0.439 |  |  | 0.139 |
| no | 32 | 77 |  | 20 | 35 | |  | 6 | 23 |  |
| yes | 71 | 84 |  | 19 | 22 | |  | 18 | 27 |  |
| Location |  |  | 0.663 |  |  | | 0.566 |  |  | 0.062 |
| Cardiac  /stomach bottom | 7 | 9 |  | 18 | 25 | |  | 3 | 7 |  |
| Gastric body | 38 | 71 |  | 7 | 12 | |  | 6 | 20 |  |
| Gastric antrum | 48 | 64 |  | 14 | 15 | |  | 17 | 18 |  |
| Stomach angle | 10 | 18 |  | 1 | 5 | |  | 0 | 5 |  |
| All the stomach | 0 | 1 |  | 0 | 1 | |  | 0 | 0 |  |
| Smoking history |  |  | 0.064 |  |  | | 1.000 |  |  | 0.076 |
| no | 85 | 117 |  | 35 | 63 | |  | 22 | 31 |  |
| yes | 18 | 46 |  | 0 | 0 | |  | 4 | 19 |  |
| Drinking history |  |  | 0.518 |  |  | | 1.000 |  |  | 0.346 |
| no | 88 | 133 |  | 35 | 63 | |  | 22 | 36 |  |
| yes | 15 | 30 |  | 0 | 0 | |  | 4 | 14 |  |
| HER2 |  |  | 0.283 |  |  | | 0.394 |  |  | 0.810 |
| positive | 4 | 13 |  | 7 | 6 | |  | 5 | 6 |  |
| negative | 92 | 139 |  | 28 | 48 | |  | 21 | 37 |  |
| Family tumor history |  |  | 0.355 |  |  | | 1.000 |  |  | 1.000 |
| no | 91 | 136 |  | 35 | 62 | |  | 22 | 43 |  |
| yes | 12 | 27 |  | 0 | 1 | |  | 4 | 7 |  |
| pTNM |  |  | <0.001 |  |  | | 0.022 |  |  | 0.003 |
| Ⅰ | 20 | 66 |  | 5 | 18 | |  | 3 | 17 |  |
| Ⅱ | 12 | 35 |  | 6 | 14 | |  | 6 | 9 |  |
| Ⅲ | 56 | 57 |  | 23 | 24 | |  | 10 | 23 |  |
| Ⅳ | 15 | 5 |  | 6 | 2 | |  | 7 | 1 |  |
| Differentiation |  |  | 0.670 |  |  | | 0.259 |  |  | 0.627 |
| Well | 0 | 2 |  | 0 | 5 | |  | 1 | 3 |  |
| Moderate | 11 | 20 |  | 11 | 12 | |  | 3 | 10 |  |
| Poor | 91 | 140 |  | 28 | 37 | |  | 22 | 36 |  |
| Signet-ring cell carcinoma | 1 | 1 |  | 1 | 1 | |  | 0 | 1 |  |
| BMI(kg/m^2^) |  |  | 0.120 |  |  | | 0.931 |  |  | 0.001 |
| <=18.5 | 18 | 15 |  | 9 | 15 | |  | 4 | 1 |  |
| 18.5~23.9 | 63 | 105 |  | 25 | 35 | |  | 21 | 29 |  |
| >=24 | 22 | 43 |  | 5 | 8 | |  | 1 | 20 |  |
| Lauren type |  |  | 0.999 |  |  | | 0.732 |  |  | 0.161 |
| Diffuse | 45 | 71 |  | 19 | 26 | |  | 2 | 15 |  |
| Intestinal | 24 | 39 |  | 9 | 17 | |  | 13 | 21 |  |
| Mixed | 32 | 50 |  | 10 | 14 | |  | 10 | 13 |  |
| Unknown | 2 | 3 |  | 2 | 1 | |  | 1 | 1 |  |
| CEA(ng/ml) |  |  | 1.000 |  |  | | 0.294 |  |  | 0.229 |
| <=5 | 94 | 150 |  | 32 | 52 | |  | 18 | 42 |  |
| >5 | 9 | 13 |  | 8 | 6 | |  | 8 | 8 |  |
| CA19-9(U/ml) |  |  | 0.099 |  |  | | 0.644 |  |  | 0.233 |
| <=35 | 89 | 152 |  | 33 | 51 | |  | 20 | 45 |  |
| >35 | 14 | 11 |  | 7 | 7 | |  | 6 | 5 |  |
| CA72-4(U/ml) |  |  | 0.828 |  |  | | 0.354 |  |  | 0.007 |
| <=6.9 | 86 | 139 |  | 36 | 47 |  | | 15 | 44 |  |
| >6.9 | 17 | 24 |  | 4 | 11 |  | | 11 | 6 |  |

**Legend**: BMI, body mass index; CA19-9, carbohydrate antigen 199; CA72-4, carbohydrate antigen 724; CEA, carcinoembryonic antigen; HER2, human epidermal growth factor receptor 2; pTNM, pathological, tumor, node, metastasis.

^a^Chi-square test was used to calculate *P* values.
