# Supplementary material for: Diagnosis and prognosis prediction of gastric cancer by high-performance serum lipidome fingerprints
Source: EMBO Mol Med. 2024 Nov 14;16(12):3089–112. doi: 10.1038/s44321-024-00169-0 (PMC11628598; doi:10.1038/s44321-024-00169-0)
Supplement: Supplementary file 8 — Table EV8 [file 44321_2024_169_MOESM8_ESM.pdf]

Table EV8. Univariate and multivariate Cox proportional hazards analyses of overall survival in GC patients of three cohorts.

Characteristics	Exploration cohort						External validation cohort						Predictive cohort					
	Univariable			Multivariable			Univariable			Multivariable			Univariable			Multivariable		
	HR	95% CI	*P* value[a]	HR	95% CI	*P* value[a]	HR	95% CI	*P* value[a]	HR	95% CI	*P* value[a]	HR	95% CI	*P* value[a]	HR	95% CI	*P* value[a]
Sex	0.696	0.406~1.191	0.186	0.957	0.469~1.952	0.904	0.680	0.332~1.395	0.293	0.892	0.345~2.310	0.814	0.617	0.234~1.622	0.327	0.346	0.057~2.105	0.249
Age	1.498	0.677~3.315	0.318	1.305	0.537~3.173	0.557	0.813	0.284~2.330	0.700	1.011	0.245~4.174	0.988		NA			NA	
Differentiation	2.648	1.044~6.719	0.040	4.777	0.796~28.676	0.087	0.940	0.534~1.658	0.832	0.654	0.311~1.374	0.262	2.324	0.656~8.229	0.191	0.752	0.137~4.138	0.744
Maximun diameter	2.838	1.623~4.966	<0.001	1.781	0.915~3.464	0.089	1.652	0.772~3.532	0.196	0.859	0.344~2.145	0.745	4.700	1.804~12.245	0.002	0.683	0.171~2.724	0.589
pTNM	10.088	4.312~23.599	<0.001	2.716	0.963~7.658	0.059	5.289	2.021~13.845	0.001	2.723	0.845~8.782	0.094	5.341	1.530~18.647	0.009	6.967	1.402~34.627	0.018
Vascular invasion	4.293	2.330~7.911	<0.001	1.703	0.851~3.407	0.133	2.230	1.075~4.627	0.031	1.280	0.509~3.221	0.600	3.083	1.086~8.759	0.035	1.293	0.321~5.214	0.718
Nerve infiltration	6.458	2.760~15.110	<0.001	2.922	0.956~8.926	0.060	1.402	0.676~2.905	0.364	0.933	0.394~2.209	0.874	2.479	0.807~7.613	0.113	0.812	0.176~3.738	0.789
HER2	1.518	0.602~3.827	0.376	1.328	0.392~4.495	0.648	1.576	0.596~4.162	0.359	0.707	0.218~2.295	0.564	1.117	0.320~3.892	0.862	1.006	0.173~5.865	0.995
Smoking history	0.686	0.345~1.364	0.282	0.769	0.278~2.129	0.613		NA			NA		0.655	0.213~2.011	0.460	2.088	0.166~26.199	0.568
Drinking history	0.805	0.380~1.706	0.572	1.072	0.419~2.737	0.885		NA			NA		0.721	0.207~2.514	0.608	1.550	0.117~20.595	0.740
Family tumor history	0.677	0.289~1.582	0.367	0.548	0.189~1.590	0.268		NA			NA		1.595	0.458~5.557	0.463	1.563	0.319~7.668	0.582
BMI	0.754	0.441~1.290	0.303	0.884	0.465~1.679	0.705	0.591	0.241~1.446	0.249	1.004	0.336~3.004	0.994	0.276	0.102~0.748	0.011	0.491	0.119~2.022	0.325
CEA	1.012	0.403~2.541	0.980	0.952	0.329~2.755	0.928	0.928	0.324~2.660	0.889	0.539	0.114~2.559	0.437	3.613	1.374~9.506	0.009	6.285	1.283~30.797	0.023
CA19-9	3.260	1.716~6.197	<0.001	1.505	0.675~3.353	0.318	3.432	1.564~7.535	0.002	2.923	1.051~8.126	0.040	3.333	1.170~9.498	0.024	3.261	0.576~18.463	0.181
CA72-4	2.401	1.323~4.357	0.004	1.790	0.863~3.714	0.118	0.579	0.176~1.909	0.369	0.800	0.215~2.977	0.740	2.197	0.770~6.272	0.141	0.670	0.115~3.915	0.657
Subtype	3.344	1.912~5.848	<0.001	2.224	1.141~4.336	0.019	3.970	1.852~8.510	<0.001	3.566	1.355~9.388	0.010	6.460	2.267~18.411	<0.001	6.386	1.676~24.336	0.007

Legend: BMI, body mass index; CA19-9, carbohydrate antigen 199; CA72-4, carbohydrate antigen 724; CEA, carcinoembryonic antigen; CI, confidence interval; GC, gastric cancer; HER2, human epidermal growth factor receptor 2; HR, hazard ratio; pTNM, pathological, tumor, node, metastasis.

[a]Wald Test was used to calculate *P* value.
