# Supplementary material for: Diagnosis and prognosis prediction of gastric cancer by high-performance serum lipidome fingerprints
Source: EMBO Mol Med. 2024 Nov 14;16(12):3089–112. doi: 10.1038/s44321-024-00169-0 (PMC11628598; doi:10.1038/s44321-024-00169-0)
Supplement: Supplementary file 9 — Table EV9 [file 44321_2024_169_MOESM9_ESM.pdf]

Table EV9. Differential expression of the SLMS metabolites in tissues and serums.

metabolites	A		B		C		D					
							GC vs HD		PL vs HD		GC vs PL	
	t statistic	*P* value[a]	t statistic	*P* value[b]	t statistic	*P* value[b]	t statistic	*P* value[b]	t statistic	*P* value[b]	t statistic	*P* value[b]
LPC 17:0	-1.18	0.268	22.566	<0.001	-8.134	<0.001	-9.352	<0.001	-7.249	<0.001	-2.051	0.042
PE O-44:6|PE O-24:2_20:4	2.642	0.027	-9.780	<0.001	8.548	<0.001	7.338	<0.001	3.412	0.001	4.517	<0.001
CAR 14:0	-2.915	0.017	4.965	<0.001	-0.579	0.563	-3.577	<0.001	-3.182	0.002	-0.314	0.754
HexCer 42:2;2O|HexCer 18:1;2O/24	-1.401	0.195	-10.396	<0.001	4.373	<0.001	3.959	<0.001	1.612	0.109	1.952	0.053
FA 28:3;O	NA		9.994	<0.001	-10.630	<0.001	-8.917	<0.001	-6.317	<0.001	-2.170	0.032
HexCer 42:2;3O	-3.810	0.004	10.487	<0.001	-1.405	0.162	-2.908	0.004	-0.864	0.389	-1.809	0.073
LPE O-14:1	NA		-9.420	<0.001	-8.010	<0.001	4.468	<0.001	-4.113	<0.001	7.087	<0.001
PE 36:4|PE 18:2_18:2	-3.063	0.014	9.724	<0.001	3.361	0.001	-0.246	0.806	-0.198	0.843	-0.008	0.994
CAR 18:1	-3.452	0.007	-8.770	<0.001	5.827	<0.001	4.051	<0.001	4.609	<0.001	-0.861	0.390
Cer 38:1;2O|Cer 18:1;2O/20:0	-5.729	<0.001	-4.257	<0.001	6.571	<0.001	3.338	0.001	2.126	0.035	1.493	0.138
TG(P) 50:2	-0.735	0.481	4.701	<0.001	-2.687	0.008	-2.096	0.038	-1.992	0.048	-0.223	0.824
FA 18:0;O	-0.739	0.479	8.102	<0.001	-7.400	<0.001	-3.839	<0.001	-5.489	<0.001	1.103	0.272
PC O-38:3	-0.132	0.898	9.495	<0.001	-2.951	0.004	-3.772	<0.001	-1.906	0.059	-1.809	0.073
FA 16:2	-2.377	0.041	7.880	<0.001	6.099	<0.001	-2.358	0.020	5.899	<0.001	-11.168	<0.001
PC O-42:6|PC O-22:2_20:4	2.047	0.071	-6.293	<0.001	0.191	0.849	3.318	0.001	-1.648	0.102	4.509	<0.001
Hex2Cer 42:2;2O|Hex2Cer 18:1;2O/	1.839	0.099	-5.243	<0.001	2.232	0.027	3.141	0.002	2.017	0.046	1.017	0.311
PE 40:7|PE 18:1_22:6	-2.762	0.022	8.069	<0.001	2.621	0.009	0.279	0.781	1.483	0.140	-1.194	0.234
LPE 20:4	-2.104	0.065	-4.161	<0.001	5.893	<0.001	2.414	0.017	2.150	0.033	0.287	0.774
PE 40:3	NA		6.990	<0.001	-2.846	0.005	-1.139	0.257	-0.651	0.516	-0.491	0.624

Legend: A, Differential expression of metabolites in the SLMS between tumor and normal tissues from 10 GC patients. B, Differential expression of 19 lipid metabolites in serums of GC patients and heathy donorss from the exploration cohort. C, Differential expression of 19 lipid metabolites in serums of GC patients and healthy donors from the external validation cohort. D, Differential expression of 19 lipid metabolites in serums between any two groups in the predictive cohort.

Abbreviations: SLMS, serum lipid metabolic signature; GC, gastric cancer; HD, healthy donor; PL, precancerous lesion;

[a]Paried t-test was used for comparing two groups.

[b]Student's t test was used for comparing two groups.
