# Supplementary material for: Diagnosis and prognosis prediction of gastric cancer by high-performance serum lipidome fingerprints
Source: EMBO Mol Med. 2024 Nov 14;16(12):3089–112. doi: 10.1038/s44321-024-00169-0 (PMC11628598; doi:10.1038/s44321-024-00169-0)
Supplement: Supplementary file 10 — Table EV10 [file 44321_2024_169_MOESM10_ESM.docx]

**Table EV10. Differential expression of 19 lipid metabolites in GC patients before surgery and after surgery compared with healthy donors.**

| Metabolite | BS vs AS | | | | BS vs HD | | | AS vs HD | | |
| --- | --- | --- | --- | --- | --- | --- | --- | --- | --- | --- |
|  | t  statistic | | | *P* value^a^ | t statistic | | *P* value^b^ | t statistic | | *P* value^b^ |
| LPC 17:0 | | -1.808 | 0.075 | | -9.675 | <0.001 | | -6.240 | <0.001 | |
| PE O-44:6  \|PE O-24:2_20:4 | | -0.272 | 0.786 | | 4.421 | <0.001 | | 5.945 | <0.001 | |
| CAR 14:0 | | -53128 | <0.001 | | -2.446 | 0.017 | | 1.640 | 0.105 | |
| HexCer 42:2;2O  \|HexCer 18:1;2O/24:1 | | -2.372 | 0.020 | | 5.953 | <0.001 | | 6.278 | <0.001 | |
| FA 28:3;O | | 0.451 | 0.654 | | -3.847 | <0.001 | | -4.467 | <0.001 | |
| HexCer 42:2;3O | | 3.789 | <0.001 | | -3.516 | 0.001 | | -7.324 | <0.001 | |
| LPE O-14:1 | | 4.801 | <0.001 | | 6.639 | <0.001 | | -1.486 | 0.142 | |
| PE 36:4\|PE 18:2_18:2 | | -1.089 | 0.280 | | -2.749 | 0.008 | | -1.947 | 0.055 | |
| CAR 18:1 | | -2.229 | 0.029 | | 4.593 | <0.001 | | 4.828 | <0.001 | |
| Cer 38:1;2O  \|Cer 18:1;2O/20:0 | | 2.686 | 0.009 | | -3.118 | 0.003 | | -4.774 | <0.001 | |
| TG(P) 50:2 | | 2.163 | 0.034 | | -3.161 | 0.002 | | -4.049 | <0.001 | |
| FA 18:0;O | | -2.166 | 0.034 | | -1.381 | 0.172 | | 1.305 | 0.196 | |
| PC O-38:3 | | 3.081 | 0.003 | | -0.571 | 0.570 | | -3.247 | 0.002 | |
| FA 16:2 | | -1.512 | 0.135 | | 0.236 | 0.814 | | 2.090 | 0.040 | |
| PC O-42:6  \|PC O-22:2_20:4 | | -2.764 | 0.007 | | 3.024 | 0.003 | | 4.620 | <0.001 | |
| Hex2Cer 42:2;2O  \|Hex2Cer 18:1;2O/24:1 | | -1.544 | 0.127 | | 3.269 | 0.002 | | 4.842 | <0.001 | |
| PE 40:7\|PE 18:1_22:6 | | -1.369 | 0.175 | | -2.032 | 0.046 | | -1.554 | 0.125 | |
| LPE 20:4 | | 0.286 | 0.776 | | 2.166 | 0.034 | | 1.802 | 0.076 | |
| PE 40:3 | | 2.840 | 0.006 | | -4.075 | <0.001 | | -6.417 | <0.001 | |

**Legend**: The analysis was based on 50 gastric cancer patients and 50 healthy donors. AS, after surgery; BS, before surgery; GC, gastric cancer; HD, healthy donor.

^a^Paired *t*-test was used for comparing groups AS and BS.

^b^Student‘s *t* test was used for comparing groups between BS and HD or AS and HD.
