# Supplementary material for: Diagnosis and prognosis prediction of gastric cancer by high-performance serum lipidome fingerprints
Source: EMBO Mol Med. 2024 Nov 14;16(12):3089–112. doi: 10.1038/s44321-024-00169-0 (PMC11628598; doi:10.1038/s44321-024-00169-0)
Supplement: Supplementary file 11 — Table EV11 [file 44321_2024_169_MOESM11_ESM.docx]

**Table EV11. The differential analysis results of enriched pathways in transcriptome and proteome.**

| pathway | transcriptome | | | | proteome | | | |
| --- | --- | --- | --- | --- | --- | --- | --- | --- |
|  | mean difference | t-statistic | P value^a^ | P-adj^a^ | mean difference | t-statistic | P value^a^ | P-adj^a^ |
| Alpha Linolenic Acid | 0.020 | 0.586 | 0.558 | 0.585 | -0.369 | -9.083 | <0.001 | <0.001 |
| Linoleic Acid Metabolism | -0.290 | -10.484 | <0.001 | <0.001 | -0.388 | -8.607 | <0.001 | <0.001 |
| Biosynthesis of unsaturated fatty acids | -0.187 | -7.175 | <0.001 | <0.001 | -0.214 | -4.475 | <0.001 | <0.001 |
| Beta Oxidation of Very Long Chain Fatty Acids | -0.084 | -2.633 | 0.009 | 0.011 | -0.275 | -4.387 | <0.001 | <0.001 |
| Glycerophospholipid Metabolism | <0.001 | 0.013 | 0.989 | 0.994 | -0.039 | -1.297 | 0.195 | 0.263 |

**Legend**: ^a^T test was used to compare two groups.
