# Supplementary material for: Diagnosis and prognosis prediction of gastric cancer by high-performance serum lipidome fingerprints
Source: EMBO Mol Med. 2024 Nov 14;16(12):3089–112. doi: 10.1038/s44321-024-00169-0 (PMC11628598; doi:10.1038/s44321-024-00169-0)
Supplement: Supplementary file 12 — Table EV12 [file 44321_2024_169_MOESM12_ESM.docx]

**Table EV12. KEGG pathway enrichment of differential genes between prognostic subtypes.**

| KEGG map | Description | Count | Odd Ratio | *P* value^a^ |
| --- | --- | --- | --- | --- |
| hsa04974 | Protein digestion and absorption | 15 | 6.485 | <0.001 |
| hsa04975 | Fat digestion and absorption | 7 | 7.249 | <0.001 |
| hsa04973 | Carbohydrate digestion and absorption | 5 | 4.737 | 0.004 |
| hsa04979 | Cholesterol metabolism | 5 | 4.365 | 0.006 |
| hsa00500 | Starch and sucrose metabolism | 4 | 4.948 | 0.008 |
| hsa04923 | Regulation of lipolysis in adipocytes | 5 | 3.839 | 0.009 |
| hsa04977 | Vitamin digestion and absorption | 3 | 5.138 | 0.020 |
| hsa00052 | Galactose metabolism | 3 | 4.174 | 0.034 |
| hsa00590 | Arachidonic acid metabolism | 4 | 2.920 | 0.048 |
| hsa00430 | Taurine and hypotaurine metabolism | 2 | 5.566 | 0.049 |

**Legend**: ^a^Hypergeometric test was used to calculate *P* value.
