# Supplementary material for: Diagnosis and prognosis prediction of gastric cancer by high-performance serum lipidome fingerprints
Source: EMBO Mol Med. 2024 Nov 14;16(12):3089–112. doi: 10.1038/s44321-024-00169-0 (PMC11628598; doi:10.1038/s44321-024-00169-0)
Supplement: Supplementary file 13 — Table EV13 [file 44321_2024_169_MOESM13_ESM.docx]

**Table EV13. The differences of druggable targets between prognostic subgroups (top20 by log_2_FC).**

| Gene | Gene long name | *P* value^a^ | log_2_FC |
| --- | --- | --- | --- |

| *MEP1B* | Meprin a Subunit Beta | <0.001 | 6.734 |
| --- | --- | --- | --- |
| *APOB* | Apolipoprotein B | <0.001 | 6.706 |
| *LY6D* | Lymphocyte Antigen 6 Family Member D | 0.005 | 5.129 |
| *APOC3* | Apolipoprotein C3 | 0.001 | 5.095 |
| *MEP1A* | Meprin A Subunit Alpha | <0.001 | 4.480 |
| *MTTP* | Microsomal Triglyceride Transfer Protein | <0.001 | 4.357 |
| *SLC2A2* | Solute Carrier Family 2 Member 2 | 0.018 | 4.121 |
| *CYP3A4* | Cytochrome P450 Family 3 Subfamily A Member 4 | <0.001 | 4.118 |
| *KLK7* | Kallikrein Related Peptidase 7 | <0.001 | 4.111 |
| *ANXA8* | Annexin A8 | <0.001 | 4.104 |
| *GSTA3* | Glutathione S-Transferase Alpha 3 | 0.005 | 4.053 |
| *SLC30A10* | Solute Carrier Familly30 Member 10 | 0.007 | 3.845 |
| *SLC26A3* | Solute Carrier Family 26 Member 3 | <0.001 | 3.817 |
| *BTNL3* | Butyrophilin Like 3 | 0.001 | 3.656 |
| *ADIPOQ* | Adiponectin, C1Q And Collagen Domain Containing | 0.019 | 3.480 |
| *CPS1* | Carbamoyl-Phosphate Synthase 1 | <0.001 | 3.403 |
| *MUC17* | Mucin 17, Cell Surface Associated | <0.001 | 3.296 |
| *SLC17A8* | Solute Carrier Family 17 Member 8 | 0.018 | 3.230 |
| *APOA1* | Apolipoprotein A1 | <0.001 | 3.130 |
| *REG4* | Regenerating Family Member 4 | 0.002 | 3.087 |
| *GLRA2* | Glycine Receptor Alpha 2 | 0.026 | -2.548 |
| *TUBA3FP* | Tubulin Alpha 3F Pseudogene | 0.006 | -2.551 |
| *TH* | Tyrosine Hydroxylase | 0.001 | -2.561 |
| *NOTUM* | Notum, Palmitoleoyl-Protein Carboxylesterase | 0.002 | -2.601 |
| *HAP1* | Huntingtin Associated Protein 1 | 0.001 | -2.651 |
| *CPA5* | Carboxypeptidase A5 | 0.020 | -2.688 |
| *FFAR1* | Free Fatty Acid Receptor 1 | 0.003 | -2.866 |
| *CPA1* | Carboxypeptidase A1 | 0.003 | -2.866 |
| *OVCH2* | Ovochymase 2 (Gene/Pseudogene) | 0.005 | -2.878 |
| *VIP* | Vasoactive Intestinal Peptide | <0.001 | -2.879 |
| *PHOX2B* | Paired Like Homeobox 2B | 0.047 | -3.286 |
| *PRLHR* | Prolactin Releasing Hormone Receptor | 0.003 | -3.352 |
| *SLC7A14* | Solute Carrier Family 7 Member 14 | 0.004 | -3.401 |
| *MAG* | Myelin Associated Glycoprotein | 0.027 | -3.644 |
| *FBN3* | Fibrillin 3 | 0.003 | -3.774 |
| *CSAG2* | CSAG Family Member 2 | 0.031 | -3.778 |
| *CARTPT* | Cart Prepropeptide | <0.001 | -4.198 |
| *HTR2C* | 5-Hydroxytryptamine Receptor 2C | 0.033 | -4.570 |
| *CHAT* | Choline O-Acetyltransferase | <0.001 | -5.388 |
| *GAST* | Gastrin | <0.001 | -6.400 |

**Legend**: log_2_FC, logarithmic scale of the ratio of SII to SI in base 2;

^a^Deseq2 was used to analyze the differences.
