# Supplementary material for: Diagnosis and prognosis prediction of gastric cancer by high-performance serum lipidome fingerprints
Source: EMBO Mol Med. 2024 Nov 14;16(12):3089–112. doi: 10.1038/s44321-024-00169-0 (PMC11628598; doi:10.1038/s44321-024-00169-0)
Supplement: Supplementary file 17 — Expanded View Figures [file 44321_2024_169_MOESM17_ESM.pdf]

Expanded View Figures

Figure EV1. The analysis of preliminary experiment data and quality control data.

(**A**) PCA on the lipid data or hydrophilic metabolite data of GC patients ($n = 28$) and healthy donors ($n = 28$). (**B**) PCA on the participant samples and QC samples showed that the QC samples were highly correlated. (**C**) Spearman's correlation coefficients between QC runs, ranging from 0.97 to 1, demonstrated the high stability and reproducibility of data. (**D**) Intensity distribution of lipid species indicated that QC samples ($n = 49$) had good consistency with participant samples in quantification of serum lipid levels; The sample numbers of GC, HD, and PL groups have been shown in Fig. 1A. (**E**) The validity of the partial least squares-discriminant analysis in Fig. 1B showed no overfitting (permutation test, $n = 1000$). Q2 measures the predictive ability of the model, while R2Y measures the goodness of fit. (**F**) The significantly changed lipids between GC patients and healthy donors. The classes of lipids are displayed in different colors. The black circle indicates 0 of the lipid level and the height of the bar represents the normalized lipid levels. The direction of bars pointing towards and away from the center represents the lipid level of healthy donors and GC patients, respectively. (**G**) The pathway enriched by the significantly changed lipid in serums (Hypergeometric test). The definitions of box plots in (**A**) and (**D**) were consistent with those in Fig. 3A,B. PCA principal component analysis, PC principal component, QC quality control, GC gastric cancer, HD healthy donor, PL precancerous lesion.

© The Author(s)

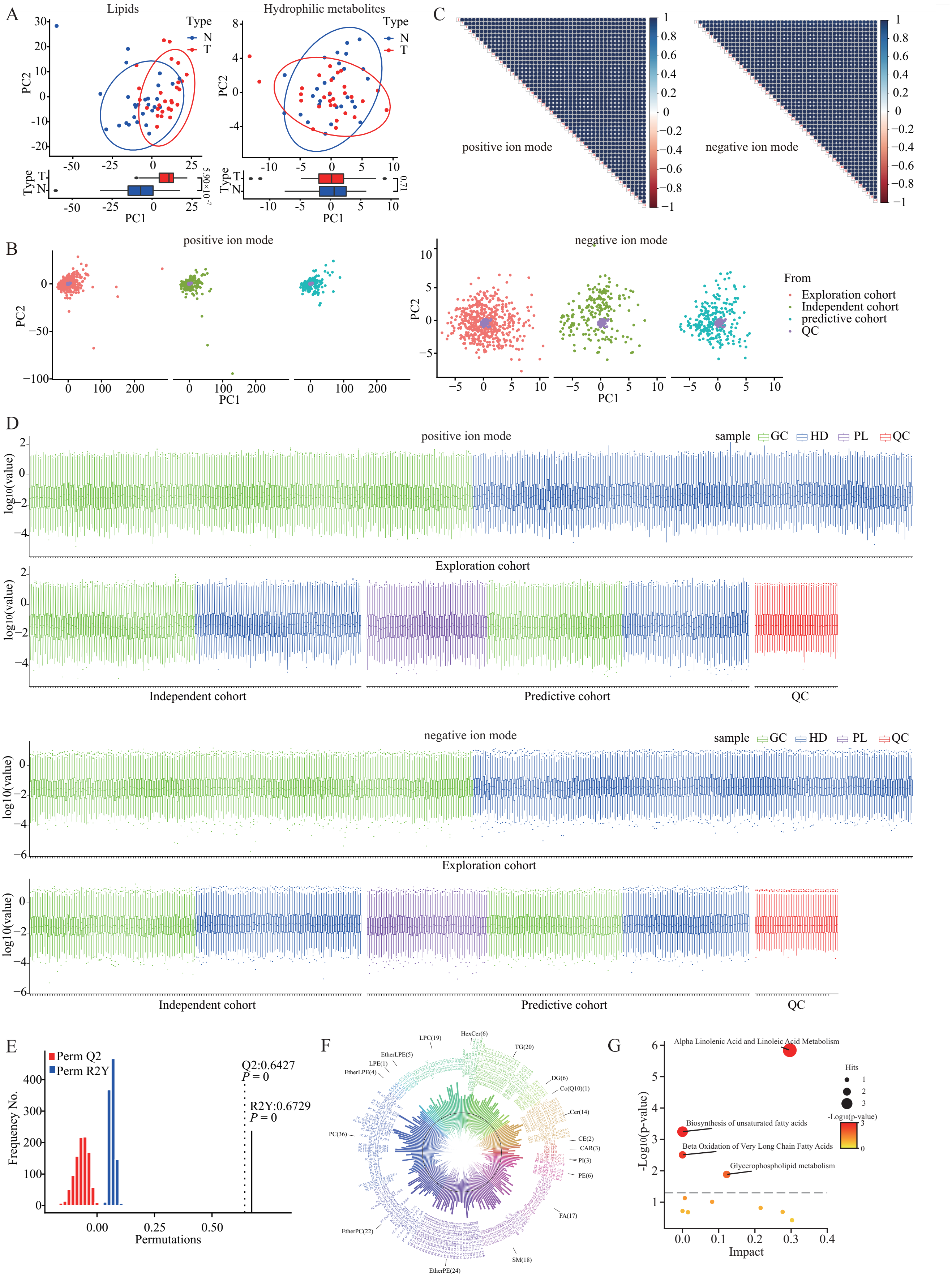
A
Lipids
Hydrophilic metabolites
Type
N
T
PC2
PC1
C
positive ion mode
negative ion mode
B
positive ion mode
negative ion mode
From
Exploration cohort
Independent cohort
predictive cohort
QC
D
positive ion mode
sample GC HD PL QC
log10(value)
Exploration cohort
Independent cohort
Predictive cohort
QC
negative ion mode
E
Perm Q2
Perm R2Y
Q2:0.6427
P = 0
R2Y:0.6729
P = 0
Frequency No.
Permutations
F
G
Alpha Linolenic Acid and Linoleic Acid Metabolism
Biosynthesis of unsaturated fatty acids
Beta Oxidation of Very Long Chain Fatty Acids
Glycerophospholipid metabolism
Hits
-Log10(p-value)
Impact

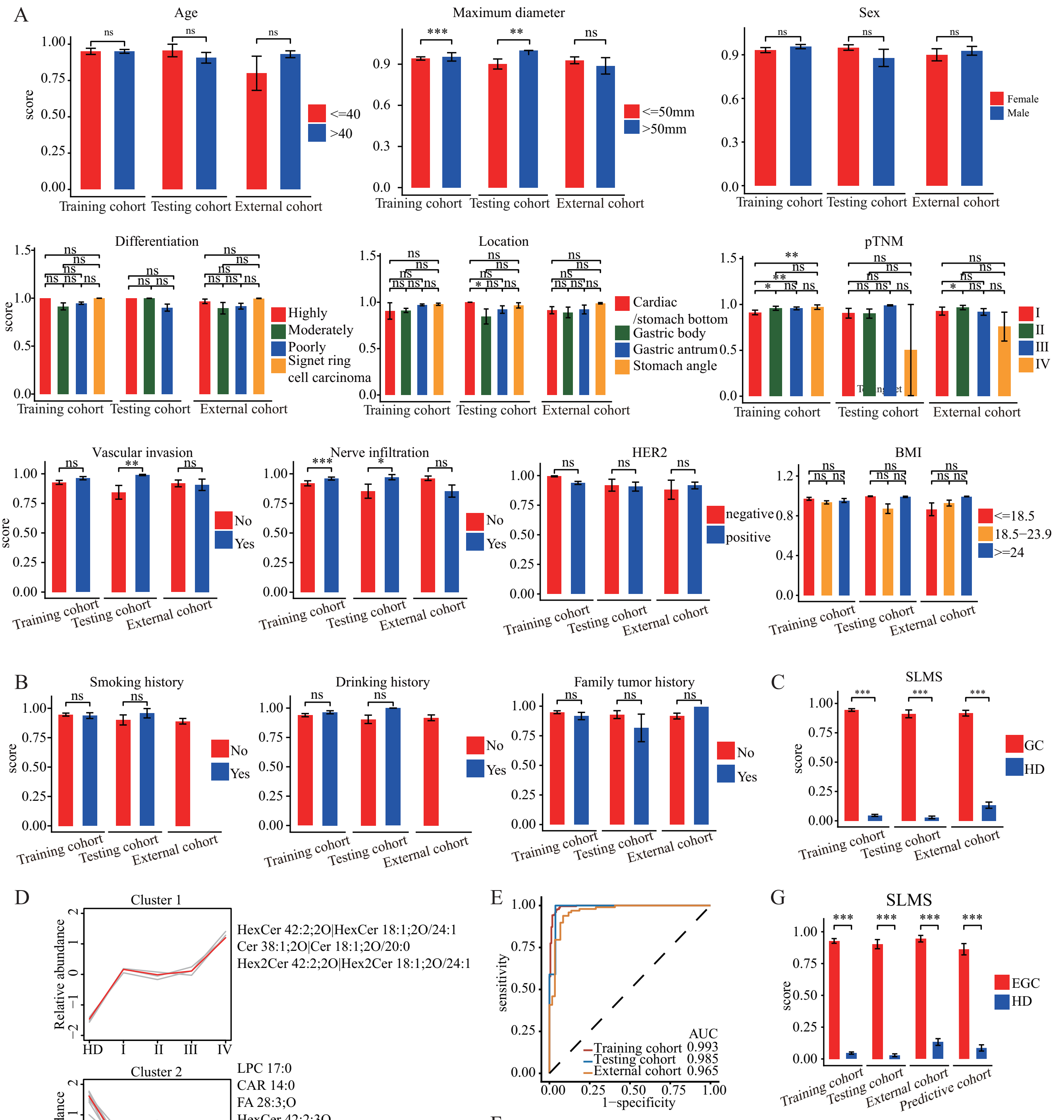

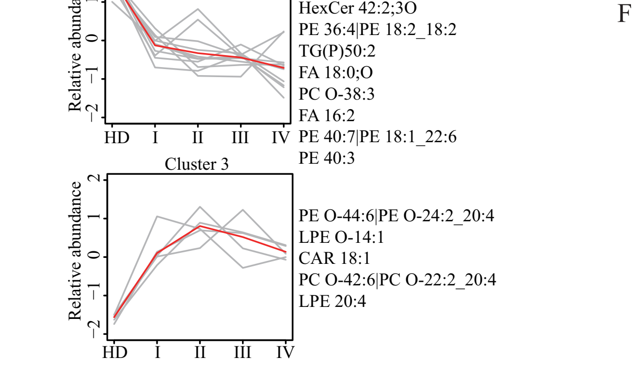

F

cohort (NTB vs HD)	Accuracy (95% CI)	Sensitivity (95% CI)	Specificity (95% CI)
Training cohort	0.965	0.952	0.974
168 vs 227	(0.941~0.981)	(0.908~0.979)	(0.943~0.990)
Testing cohort	0.970	0.926	1.000
27 vs 39	(0.895~0.996)	(0.757~0.991)	(0.910~1.000)
External validation cohort	0.911	0.932	0.898
59 vs 98	(0.855~0.950)	(0.835~0.981)	(0.820~0.950)

© The Author(s)

◀ **Figure EV2. The influence factor of the score of SLMS.**

(**A**) The SLMS scores of GC patients were compared between different stratification of age, maximum diameter, sex, differentiation, location, pTNM, vascular invasion, nerve infiltration, HER2 and BMI in the training, testing and external validation cohorts. (**B**) The SLMS scores of GC patients were compared between different stratification of smoking history, drinking history and family tumor history in the training, testing, and external validation cohorts. (**C**) The difference between the SLMS score of GC patients and that of HDs in the training, testing, and external validation cohorts. (**D**) Mfuzz clustering of lipid trajectories during GC progression using 19 lipids according to the lipid changes' similarity. Lipids in each cluster are presented on the side. HD, healthy donor. (**E**, **F**) The diagnostic performance of SLMS when used in detecting GC patients with negative CEA, CA19-9, and CA72-4. (**G**) The difference between the SLMS score of EGC patients and that of HDs in the training, testing, external validation, and predictive cohorts. *P* values were determined by Wilcox test and Data presented as the mean ± S.D. (**A**, **B**, **C**, **G**). CA19-9 carbohydrate antigen 199, CA72-4 carbohydrate antigen 724, CEA carcinoembryonic antigen, CI confidence interval, EGC early-stage gastric cancer, GC gastric cancer, HD healthy donor, NTB negative for three biomarkers, SLMS serum lipid metabolic signature, ns non-significant; ****P* < 0.001; ***P* < 0.01; **P* < 0.05. Source data are available online for this figure.

 © The Author(s)

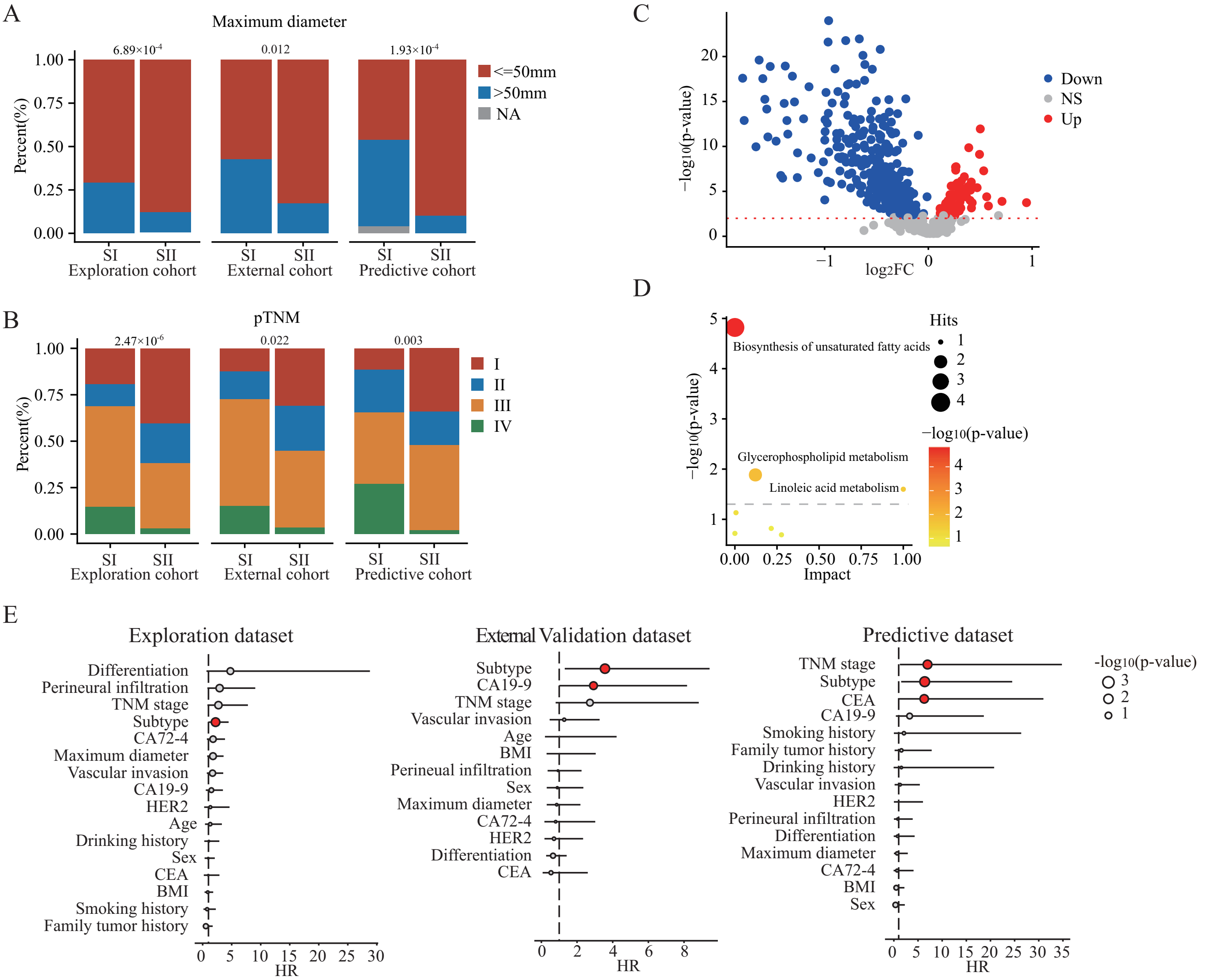

Figure EV3. Characterization of GCPS.

(A) The overlap between GCPS and maximum diameter. **(B)** The overlap between GCPS and pTNM stage. **(C)** Volcano plot comparing different lipids between SI and SII. **(D)** Enrichment analysis of different lipids between SI and SII. Hits are indicated by the size of the circle and significance is indicated by the color of the circle. **(E)** Multivariate Cox proportional hazards analyses of OS in patients with gastric cancer in three cohorts. The circles in red color indicated the *P* value was less than 0.05. *P* values were determined by Chi-square test (**A**, **B**), Wilcox test (**C**), Hypergeometric test (**D**), and Wald test (**E**). GCPS gastric cancer prognostic subtype, NS non-significant, OS overall survival, pTNM pathological Tumor-Node-Metastasis.

© The Author(s)

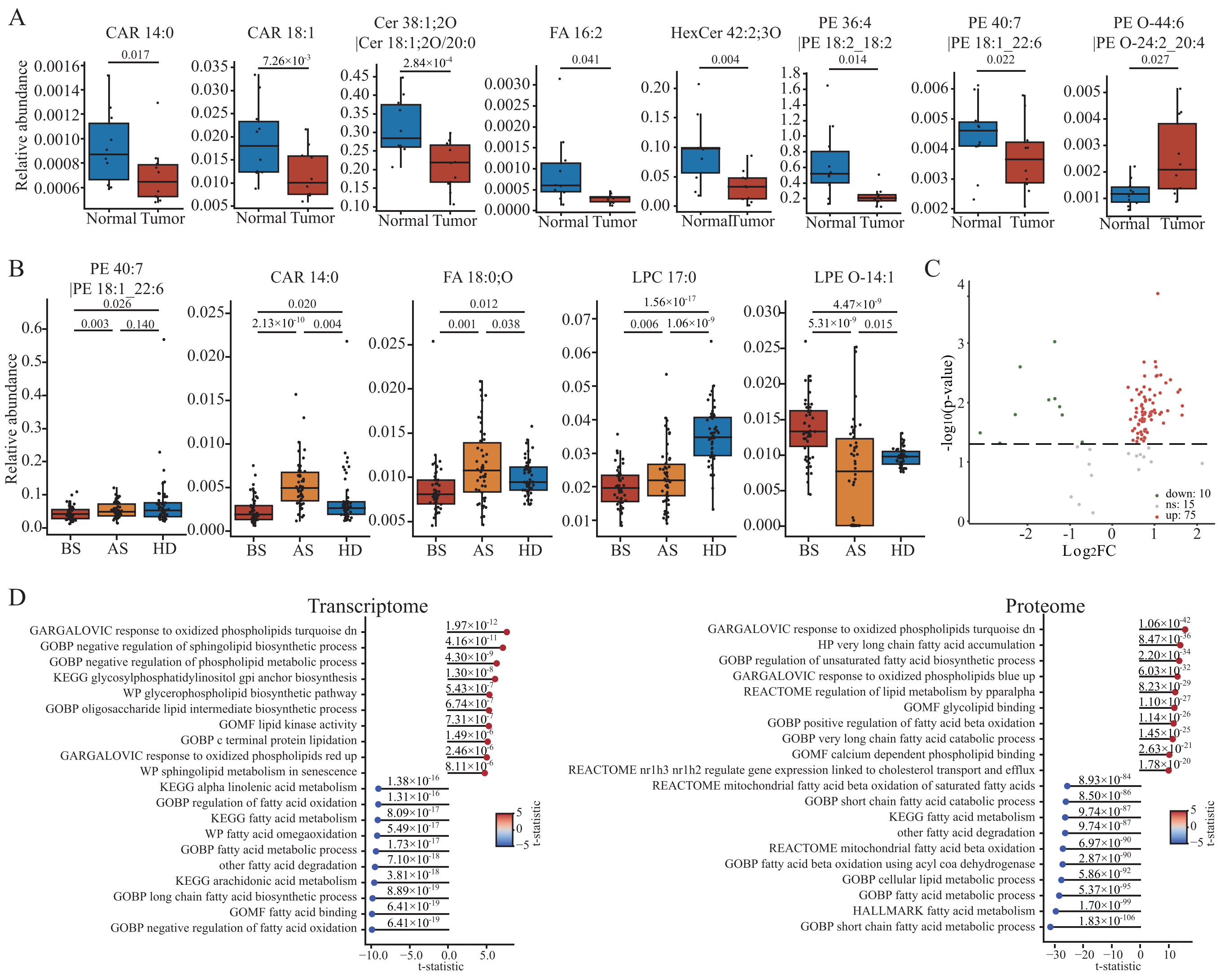

Figure EV4. Analysis of the metabolites in SLMS.

(**A**) The significantly changed metabolites between gastric cancer ($n = 10$) and normal tissues ($n = 10$). (**B**) Metabolites that partially or completely return to normal levels after surgery ($n = 50$ per group). (**C**) Volcano plot showing the lipids that were differentially expressed between GC and normal regions. (**D**) Top 10 lipid-related metabolic pathways highly expressed in cancer tissues and normal tissues according to the transcriptome and proteome analysis. *P* values were determined by T test (**A–D**) and adjusted via the Benjamini-Hochberg procedure (**D**). The definitions of box plots in (**A**) and (**B**) were consistent with those in Fig. 3A,B. AS after surgery, BS before surgery, HD healthy donor, SLMS serum lipid metabolic signature.

 © The Author(s)

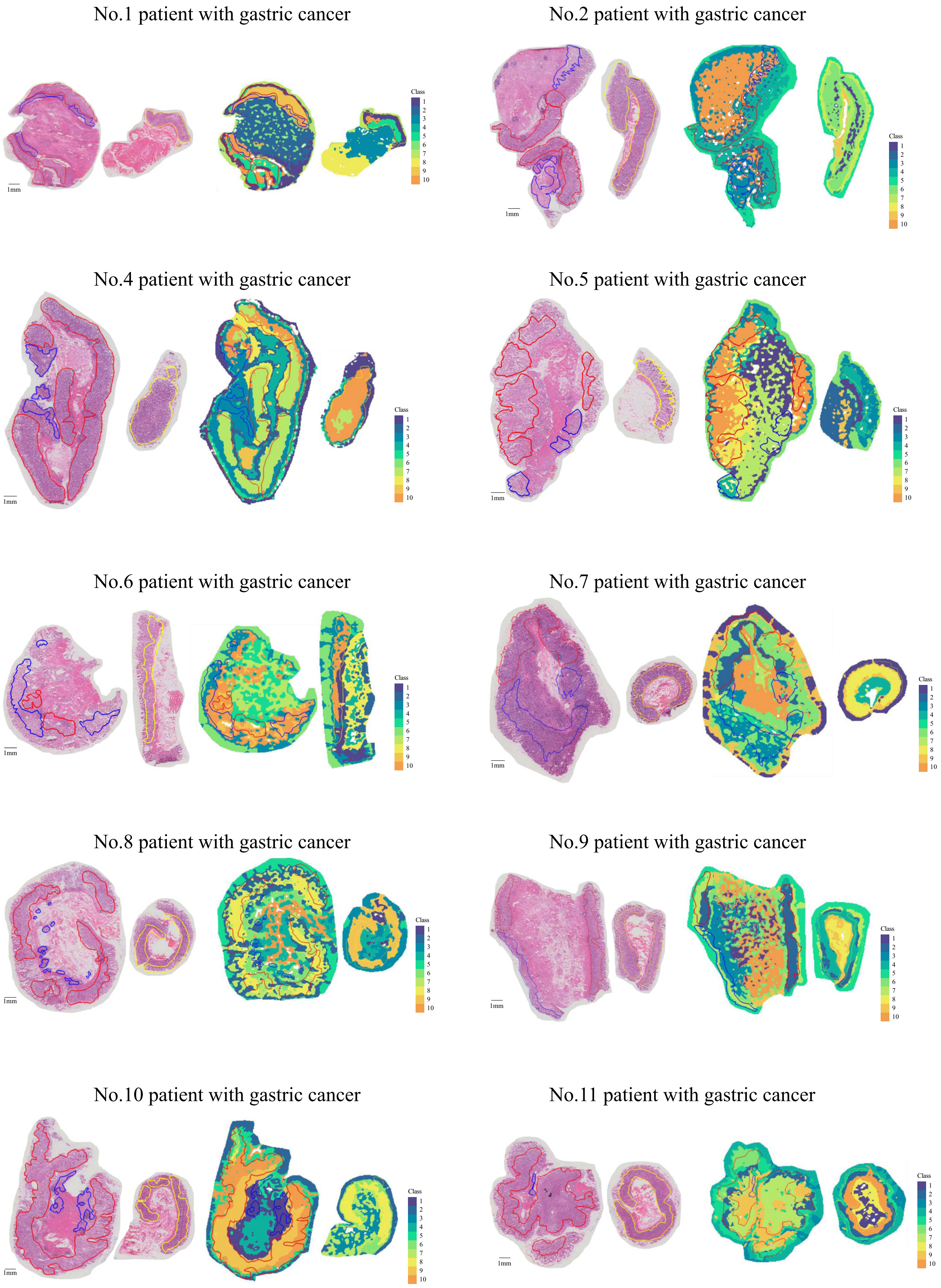
No.1 patient with gastric cancer
No.2 patient with gastric cancer
No.4 patient with gastric cancer
No.5 patient with gastric cancer
No.6 patient with gastric cancer
No.7 patient with gastric cancer
No.8 patient with gastric cancer
No.9 patient with gastric cancer
No.10 patient with gastric cancer
No.11 patient with gastric cancer
1mm
Class
1
2
3
4
5
6
7
8
9
10

◀ **Figure EV5. The global metabolic landscape of patients with gastric cancer.**

H&E stain image and metabolite-driven segmentation of contiguous gastric cancer tissue sections. Scale bar = 1 mm. The blue, red, and yellow areas represent tumor, paratumor, and normal regions, respectively.

 © The Author(s)
